# African laboratory medicine in the time of COVID-19

**DOI:** 10.4102/ajlm.v9i1.1447

**Published:** 2020-12-21

**Authors:** Iruka N. Okeke

**Affiliations:** 1 Department of Pharmaceutical Microbiology, Faculty of Pharmacy, University of Ibadan, Ibadan, Nigeria

**FIGURE 1:**
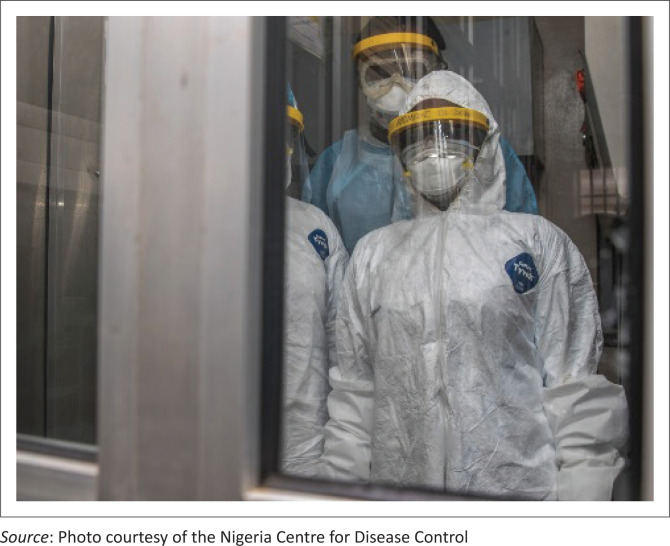
In the time of COVID-19. The importance of laboratory medicine has been made more visible by COVID-19 than at any time this century.

2020 has been a turbulent year but one that fixed the importance of laboratory medicine in the eye of the global public. Public health experts’ worst fears surrounding a hypothetical ‘Agent X’ that spurred thousands of calls for ‘pandemic preparedness’ in the last half-decade have become eerily true and the importance of ‘testing’ is now broadly acknowledged. By the end of the first quarter of 2020, following its emergence in Wuhan, China, Agent X was named severe acute respiratory syndrome coronavirus 2 (SARS-CoV-2); it had caused infections in almost every country, and one of the worst pandemics of all time was underway (Figure 1). Instituting SARS-CoV-2 testing rapidly and reliably was one of the pivotal distinguishers between countries that got a handle on their coronavirus disease 2019 (COVID-19) epidemics and those that did not.

No country in the world was ready by the time COVID-19 overwhelmed each national surveillance and health system. Across Africa, pandemic response planning began early and, as SARS-CoV-2 entered our continent relatively late, most African countries were able to deploy testing before the virus arrived.^1,2^ To their tremendous credit, African countries leveraged this testing advantage to implement pandemic infection control policies that likely combined with as yet unknown biological features to yield what are still now relatively temperate versions of COVID-19 devastation. African countries have also continued to grow testing capacity so that, while we are not the necessary step ahead of the virus, most countries in Africa are, to some degree, keeping pace with it.

National epidemic responses depend on very local activities. Published this year in the *African Journal of Laboratory Medicine* (AJLM) is an exemplary local response to a COVID-19 crisis in an African SARS-CoV-2 testing laboratory. The crisis was a laboratory outbreak that not only endangered the medical laboratory scientists, but also disabled the testing service when the need was greatest.^3^ Among other things, the affected laboratory implemented heightened infection prevention and control measures and increased physical distancing. It also increased automation of sample processing, which reduced contact between staff and infected specimens, and will improve operations in the years to come. As another article from Skosana et al.^4^ demonstrates, workplace infections are an ever-present risk in laboratories and so lessons learned from this pandemic will have broad application.

At the patient level, a significant proportion of COVID-19 survivors live for months with disabling sequelae in ‘long-COVID’; the ultimate prognosis for this new disease remains unclear.^5^ COVID-19 also has unclear long-haul implications on communities, on countries and on healthcare systems and their laboratories. The pandemic is a frontal challenge to weak systems, but it also offers opportunities to develop them and build resilience, if the response is implemented with this in mind.^6^ For example, future procurement could be templated on more generally applicable network approaches to procurement and supply chain management outlined by Williams et al. for HIV, forestalling the procurement crises experienced in Africa with COVID-19 testing and personal protective equipment supplies.^7,8^

SARS-CoV-2 is not the only epidemic pathogen that circulated in Africa in 2020. The Democratic Republic of Congo declared a 21-month Ebola outbreak that ended on 14 May 2020, but on 01 June 2020, a new outbreak began. Nigeria has worked to contain Lassa fever virus epidemics for most of the duration of the COVID-19 pandemic. While these three feared viruses ravage, measles, cholera and other epidemics are also in play.^8^ One of the things that has become increasingly visible in the course of these epidemics is the central role laboratory medicine must play to contain them. Volume 9, Issue 1 of AJLM goes beyond chronicling a broad range of infectious disease catastrophes to outlining lessons learned that will strengthen health systems.^3,9,10,11,12,13,14,15,16,17,18^ While most pandemic activity has focused on reverse transcription polymerase chain reaction and other forms of testing to support the identification of cases and transmission chains,^10^ other domains of laboratory medicine have made important contributions that shed more light on the pathogenesis of the disease, and therefore how best to improve treatment.^19,20^

As clinical and laboratory health workers and the scientific community engaged in discoveries to combat the pandemic, another equally frenetic behind-the-scenes response took place in editorial offices as we, gratefully helped by volunteer reviewers, struggled under the mound of COVID-19 submissions to bring to the fore discoveries that are most worthy of priority and long-term attention. In mid-May, relatively early in the pandemic, a *Science* commentary reported that COVID-19 researchers and policymakers were becoming overwhelmed by the literature in this brand new field with a 20-day doubling time on the number of articles indexed by major databases.^21^ By early November, that curve had flattened somewhat but, still, over 70 000 COVID-19 articles were indexed in PubMed or posted on pre-print servers, awaiting assessment. The flood has spurred the creation of artificial intelligence approaches for curating the literature.^21^ But the bulk of the work required to review, improve and present these works to the drowning community is done manually by editors and volunteer reviewers. Unsurprisingly, the pandemic has seen highly publicised discourse on research article quality, as well as some very high-profile retractions. The strain on peer review has also led to some misleading information driving COVID-19 policy, prevention and therapeutics, some of which have been amplified by influential non-scientists. Among the many lessons learned on the fly in this pandemic is that health crises bring an influx of emergency-care patients, specimens requiring immediate testing and a flood of literature. Resilience is needed as much in scientific publishing as in health systems. Pandemic preparedness must include plans for rapidly but effectively sifting through the literature flood to retrieve the knowledge most likely to reign in the emergency, which is the only way to stop the overwhelm on both fronts.

As a regional journal addressing laboratory medicine on a continent that has seen multiple epidemics this year, we at AJLM quickly found that our standard workflows would not manage submissions fast and rigorously enough for a helpful pandemic response. At the same time, it was important for us to continue to process manuscripts across the journal’s scope. We did manage to ensure that the journal could contribute in valuable ways towards addressing the pandemic while at the same time ensuring that important articles not focused on the pressing problem of COVID-19 were published. Indeed, by responding to COVID-19 but not succumbing to ’COVIDdisation’,^22^ we observed that articles submitted before the pandemic was declared were key to the COVID-19 response. Among these are the *Future of Diagnostics* special issue article by Preiser and Van Zyl^23^ on pooled testing, with a focus on HIV, which included critical knowledge for aligning test-and-trace COVID-19 needs with the extreme resource limitations that almost every country has seen associated with polymerase chain reaction testing. Additional articles in that special issue also have direct relevance to strengthening testing capabilities in the pandemic.^24,25^

To meet the double demands of maintaining peer review and production of regular articles, the submission rate of which continues to rise, and processing pandemic submissions, the pandemic pushed us to alter our operations. AJLM’s new normal allows for a regular submission track, and a new fast track for articles containing knowledge that could be applied to the ongoing COVID-19, Lassa fever and Ebola haemorrhagic fever outbreaks. This year, these ran in addition to the call to our special issue (Volume 9, Issue 2) on the future of diagnostics, which was guest edited by Timothy Amukele, Noah Fongwen and Rosanna Peeling.^26^ Our fast track remains open and will include the many excellent articles we are processing now for Volume 10.

Altogether, AJLM received 47 submissions through its viral epidemic fast track between April 2020 when we opened the track and October of the same year. Only two of these epidemic articles addressed Lassa fever, and none addressed Ebola haemorrhagic fever, pointing to the disproportionately low scientific activity on local African epidemics. So far, just 10 of the COVID-19 fast-tracked articles have been accepted. This acceptance rate compares with that for regular submissions but is unexpectedly low, because we expected predominantly pressing issues worthy of publication to come via the fast track. Similar high submission, low acceptance rates have been reported by editors of other journals.^27^

Do the low acceptance rates make all the extra hard work done on pandemic submissions by the editorial offices worth it? This question is important to us, because all three epidemics are still in play, our journal’s overall submission rate has continued to rise and our fast track is still open. The answer is undoubtedly yes. Firstly, by overseeing the peer review of the manuscripts we received, we did help to shield frontline health workers and policymakers from the even larger deluge of COVID-19 preprints that they would otherwise have had to navigate. This is an important function of peer review in general. Secondly and more pointedly, we are proud to be publishing key articles that influence the course of the pandemic, particularly on the African continent, where the dynamics are different, the response, while variable, is largely commendable and resources are severely limited. Given the sacrifices made in so many areas towards containing this pandemic, this is the least we could do.
